# Temporal and spatial expression of cuticular proteins of *Anopheles gambiae* implicated in insecticide resistance or differentiation of M/S incipient species

**DOI:** 10.1186/1756-3305-7-24

**Published:** 2014-01-15

**Authors:** Laura Vannini, Tyler W Reed, Judith H Willis

**Affiliations:** 1 Department of Cellular Biology, University of Georgia, 724 Biological Science Building, 30602, Athens, GA, USA

**Keywords:** Cuticle, Incipient species, Insecticide resistance, TEM immunolocalization, *In situ* hybridization

## Abstract

**Background:**

Published data revealed that two of the 243 structural cuticular proteins of *Anopheles gambiae*, CPLCG3 and CPLCG4, are implicated in insecticide resistance and a third, CPF3, has far higher transcript levels in M than in S incipient species. We studied the distribution of transcripts for these three genes in the tissues of *An. gambiae* and the location of the proteins in the cuticle itself to gain information about how these cuticular proteins contribute to their important roles. Our data are consistent with CPLCG3/4 contributing to a thicker cuticle thus slowing penetration of insecticides and CPF3 possibly having a role in the greater desiccation tolerance of the M form.

**Methods:**

Using RT-qPCR, we established the temporal expression of the genes and by *in situ* hybridization we revealed the main tissues where their mRNAs are found. Electron microscopy immunolocalization, using secondary antibodies labeled with colloidal gold, allowed us to localize these proteins within different regions of the cuticle.

**Results:**

The temporal expression of these genes overlaps, albeit with higher levels of transcripts from *CPF3* in pharate adults and both *CPLCG3* and *CPLCG4* are higher in animals immediately after adult eclosion. The main location of mRNAs for all three genes is in appendages and genitalia*.* In contrast, the location of their proteins within the cuticle is completely different. CPF3 is found exclusively in exocuticle and CPLCG3/4 is restricted to the endocuticle. The other *CPF* gene expressed at the same times, *CPF4*, in addition to appendages, has message in pharate adult sclerites.

**Conclusions:**

The temporal and spatial differences in transcript abundance and protein localization help to account for *An. gambiae* devoting about 2% of its protein coding genes to structural cuticular proteins. The location of CPLCG3/4 in the endocuticle may contribute to the thickness of the cuticle, one of the recently appreciated components of insecticide resistance, while the location of CPF3 might be related to the greater desiccation resistance of the M form.

## Background

Structural cuticular proteins (CPs), chitin and lipids are the major components of the insect cuticle, the exoskeleton, as well as the cuticle that lines some internal structures such as the foregut, hindgut, tracheal system and apodemes. The 243 CPs that have been annotated for *Anopheles gambiae* comprise close to 2% of all its protein coding genes. They have been classified into a dozen distinct protein families [1,2]. Sequence domains, homology models and experimental work revealed that members of some CP families contribute to the cuticle by binding chitin; the function of others is not known. Three CPs deserve particular attention because of reported differential expression in adults in important comparisons: AgamCPF3, AgamCPLCG3 and AgamCPLCG4. Hereafter, since we will only be discussing CPs from *An. gambiae*, the Agam prefix will not be used. These genes belong to two different CP families. The CPF family has four members, two of which (CPF3 and CPF4) are only expressed in pharate adults and adults [3]. (The pharate stage begins when the epidermis has retracted from the old cuticle and has started forming the new cuticle of the next stage.) CPF1 and CPF2 are primarily expressed in larvae and pharate pupae [3]. The CPLCG3 family has 27 members with different members expressed at different times during development [4].

*CPF3* has the greatest difference in mRNA levels of transcripts in M and S incipient species of *An. gambiae* based on microarray data and confirmed with RT-qPCR on 3-d-old virgin females [5]. These incipient species are forms that only hybridize in a limited region of their range [6]. Of the five genes that were selected for RT-qPCR analysis, CPF3 was the only one with more abundant transcripts in M than in S, and the difference first found in laboratory strains was confirmed with three distinct natural populations. In these, the difference was only about 3-fold compared to the 27-fold difference in the laboratory strains [5]. Recombinant CPF3 does not bind chitin [3], and a homology model shows that the *Drosophila* pheromone 7,11-HD (7(Z), 11(Z)-heptacosadiene) would fit its binding pocket [7]. This information led to the suggestion that CPF3 might be localized in the epicuticle where it could present a contact pheromone [5,7].

**Figure 1:**
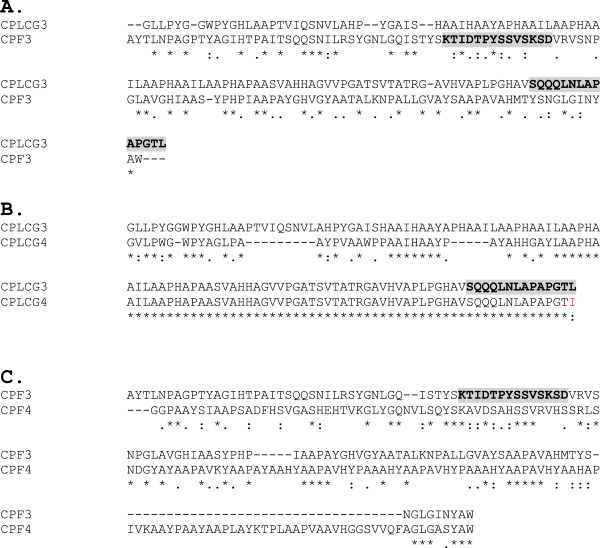
**Alignment between mature proteins using ClustalW. (A)** CPLCG3 compared to CPF3 (12% identity). **(B)** Alignment between CPLCG3 and CPLCG4 (79% identity). **(C)** Alignment between CPF3 and CPF4 (28% identity). The peptides used to raise the antibodies are in bold and highlighted. These CP genes are also known as: CPLCG3 [VectorBase: AGAP008446]; CPLCG4 [VectorBase: AGAP008447]; CPF3 [VectorBase: AGAP004690]; CPF4 [VectorBase: AGAP000382].

*CPLCG3* and the very similar *CPLCG4* (Figure 1B, Additional file 1) have been implicated in insecticide resistance in two species of *Anopheles*, because they are among the five genes that show over two-fold higher transcript levels in pyrethroid-resistant compared to pyrethroid-sensitive mosquitoes [8,9]. (CP gene names used in those papers were not the definitive ones; they are correct in [10]).

Our published studies with RT-qPCR showed that *CPF3* has significant expression first seen in pharate adults and persisting into young adults [3]. *CPLCG3* and *CPLCG4* also have highest transcript levels at those times, although the levels in young adults are higher than in pharate pupae [4]. Here we report that *CPLCG3/4* are also similar to *CPF3* in the tissues in which transcripts are found, even though they have been implicated in serving distinct roles in *Anopheles*. The amino acid sequence of CPF3 is not at all similar to CPLCG3 or CPLCG4 (Figure 1A, B). We also examined CPF4, while not implicated in insecticide resistance or M/S differences, it has sequence regions (Figure 1C) and temporal patterns of expression similar to that of CPF3, unlike the other two members of the CPF family that have transcripts primarily in pharate and young pupae [3].

While data are accumulating on the spatial distribution of individual CPs across the insect body, there is little information on localization within the cuticle itself. Electron microscopic (EM) immunolocalization has been carried out, but the proteins against which the antibodies, both polyclonal and monoclonal, had been raised were either extracts of the whole cuticle or isolated electrophoretic bands without sequence information (reviewed in [2]). We have begun to remedy this deficiency by using secondary antibodies, labeled with colloidal gold, to detect antibodies raised against specific cuticular proteins. Our focus has been on CPF3 and CPLCG3 and CPLCG4 given the importance of these specific CPs. First, we confirmed the temporal expression patterns of the selected CPs with RT-qPCR and then learned their spatial localization in tissues via *in situ* hybridization. Finally, we examined their localization in the cuticle itself using immunolocalization on EM sections.

The data we obtained provide insight into the precise roles these proteins may serve, as well as why *An. gambiae* devotes so many genes to structural cuticular proteins.

## Methods

### Mosquito rearing

The colony of *An. gambiae* (G3 strain, reported to be of the S form) was maintained at 27°C in a 14/10hL/D photoperiod (except for those used for Additional file 2 where conditions are given in the legend). Larvae were fed ground Koi food (Foster and Smith Aquatics), and adults had access to an 8% fructose solution. To obtain developmentally synchronized animals, pupae were collected at hourly intervals, separated by sex and maintained in small groups until they reached the desired age. Adults were collected on the morning after emergence (d 0) and kept in cages in a humidified insectary until used.

### *In situ* hybridization

*In situ* hybridization was carried out on 4 μm sections of paraformaldehyde-treated mosquitoes processed by the Histology Laboratory at the University of Georgia, College of Veterinary Medicine. The original probe for CPLCG3 is likely to hybridize to CPLCG4, so we designed additional probes in the 3′UTR for each of these genes (see Additional file 1B). No differences were seen in hybridization patterns among these three probes. Probes for CPF3 and CPF4 should be unique (see Additional file 1A). Details on probe construction are in [11]. Probes were labeled with dig (digoxigenin) and visualized after a 2–48 h exposure to NBT (nitro-blue tetrazolium chloride) and BCIP (5-bromo-4-chloro-3′- indolyphosphate p-toluidine). The procedure followed was a slightly simplified version of an EXIQON protocol (http://www.exiqon.com/ls/documents/scientific/edc-based-ish-protocol.pdf) and is described in detail in [11]. We carried out a limited number of hybridizations with sense probes, and found no hybridization. Also, treatment of sections with RNase prior to probe hybridization abolished hybridization to tissue but not the artifactual hybridization to the lens and cast pupal cuticle [11].

### RT-qPCR

We added some additional data to that already published [3,4] following their procedures with primers described in those papers that had been checked for efficiency and verified to amplify only a single gene (see Additional file 3). We used Bio-Rad’s MyiQ Real-Time PCR Detection System. All reactions were carried out in triplicate (technical replicates) in a 20 μl reaction containing 5 μl of 1/100 diluted cDNAs (equivalent to starting with 7.5ng of total RNA), 250 nM of each primer, and 10 μl iQ SYBR® Green Supermix (Bio-Rad). PCR conditions were 95°C for 3 min followed by 40 cycles of 95°C for 15s and 57°C for 1 min. We used 5 biological replicates (groups of three animals or three parts) for cDNA preparations. Data were normalized to *RpS7* [VectorBase: AGAP010592]*.* Different conditions and the Bio-Rad’s CFX Connect Real Time System were used for Additional file 2 and are described in the legend.

### Antibody production

Antigenic peptides were identified in our laboratory using Abie Pro 3.0 (http://www.changbioscience.com/abie/abie.html). Peptide synthesis and polyclonal antibody production were carried out by GenScript. The colloidal-gold conjugated secondary antibodies (Sigma) were 10 nm goat-anti-mouse and 5 nm goat-anti-rabbit.

The peptide against which the rabbit antibodies were raised for CPLCG3 differs only in the last amino acid from CPLCG4 (L in CPLCG3, I in CPLCG4) (Figure 1B). Hence we assume it is detecting both proteins. The peptide used for CPF3 (Figure 1A, C) was unique for that protein and the antibody was raised in mice.

It is unlikely that the antibodies will react with other cuticular proteins based on sequence differences or because the corresponding transcript is absent at the time the proteins would be secreted. The one exception is CPLCG5 that might be detected by the CPLCG3/4 antibody, although its single aa difference is inside the peptide. *In situ* hybridization revealed that it is expressed in the same tissues as CPLCG3 and CPLCG4 [11]. Details on sequence and expression of potential off-target sequences are in Additional file 4.

### Western blots

Proteins from homogenized whole bodies of mosquitoes (8-d-old) and legs (3-d-old) were extracted in 8M urea, 0.1M NaCl, 0.01M Tris, pH8.0, with protease inhibitors (cOmplete, Mini, Roche). Proteins were separated on 4-20% SDS-PAGE (Bio-Rad) with a Tris-glycine running buffer (2M glycine, 0.25 mM Tris, 1% SDS) and transferred to polyvinylidene fluoride (PVDF) (Millipore) filters. Filters were blocked with 3% bovine serum albumin (BSA) in PBST (PBS+0.1% Tween-20) for 30 min at room temperature and then incubated with anti-CPF3 (1:1,000 dilution) or anti-CPLCG3/4 (1:30,000 dilution) antibodies in PBST−1% BSA for 1 h at room temperature. After four washes with PBST (15 min at room temperature), filters were incubated with anti-mouse or anti-rabbit secondary antibodies that were conjugated to peroxidase at a dilution of 1:20,000 in PBST−1% BSA for 30 min at room temperature. Finally, filters were developed with the Western blot Chemiluminescence Reagent Plus Kit (Renaissance) and exposed to X-ray films. As controls, blots were processed in the same way without the primary antibody incubation step. Anti-CPF3 was used with proteins extracted from legs because of the unexplained high background that this antibody showed on proteins extracted from the whole body.

### Electron microscopy

The legs of pharate adults (24 h after pupation, a few hours before eclosion) and 1-d-old and 8-d-old adults were dissected. The fixation, dehydration and embedding steps were performed following [12], introducing some modifications for better integrity of mosquito cuticle. Tissues were fixed in 4% formaldehyde, 0.3% glutaraldehyde+4% sucrose in phosphate buffer 1X (PBS) (pH7.4) overnight at 4°C. Samples were rinsed three times in PBS+4% sucrose (5 min). All the subsequent steps were performed with continuous shaking at room temperature. The samples were dehydrated in a graded ethanol series: 30% ethanol-4% sucrose, 50%, 70% and 95% ethanol (10 min, each). Samples were infiltrated in 1:1 (v:v) and 1:2 (v:v) 95% ethanol:LR White resin (Electron Microscopy Sciences) and then kept in pure LR White (2 h, each), followed by an overnight change and a final change (2 h) of the resin. Samples were embedded in polyethylene capsules (that had been dried at 50°C) and covered with fresh resin. We used bottle-neck capsules, size 00 with a narrow chamber at the bottom (Polysciences) and inserted the legs vertically. Polymerization was carried out without shaking at 55°C for two d in N_2_. (This was done in a Modular Incubator Chamber, Billups-Rothenberg). Ultrathin sections (~50 nm) were cut using a diamond knife (Diatome) with a MTX ultramicrotome (Boeckeler) and placed on 200 mesh nickel grids. The sections were examined in a JEM-1210 transmission electron microscope (JEOL USA) at 120kV. The images were captured with an XR41C Bottom-Mount CCD Camera (Advanced Microscopy Techniques).

### EM Immunocytochemistry

We used results from *in situ* hybridization and RT-qPCR [3,4] to select the tissues for EM immunolocalization. Thus, the distribution of CPF3 and CPLCG3/4 was evaluated in legs of pharate adults and 1-d, and 8-d-old adults. Antibodies were diluted in 0.5M NaCl, 0.1% BSA, 0.05% TWEEN 20 and 5% FBS as follows: CPF3 (1:500), CPLCG3/4 (1:20,000), and the colloidal-gold conjugated secondary antibodies (1:50). As a negative control, sections were incubated with the pre-immune serum from the same animals from which the GenScript antibodies had been obtained. All treatments were carried out in 30 μl drops placed on parafilm in a covered Petri dish (150x15 mm). The grids with sections were incubated face down on drops of PBS (5 min), block solution (5% BSA, 2% goat serum in PBS) (30 min), primary antibody (overnight), PBS (10 min, 3X), block solution (30 min), secondary antibody (1h), PBS (10 min, 2X) and deionized water (10 min, 2X). All steps were performed at room temperature except the incubation of the primary antibody/pre-immune serum that was performed at 4°C.

## Results and discussion

### Transcript abundance

**Figure 2:**
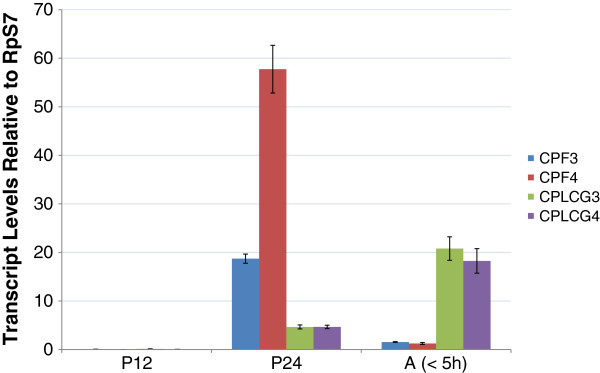
**RT-qPCR analysis of *CPF3*, *CPF4 *and *CPLCG3, CPLCG4*.** Stages examined were: pupae 12 h after pupation (P12), pharate adults (P24) and young adults (<5 h). Mean±SEM are shown. For each gene, differences between P24 and young adult are statistically significant (p≤.0001).

Temporal expression of these four genes had been monitored previously [3,4]. In order to be able to compare transcript levels on the same preparations of cDNA, we repeated these measurements with fresh material (Figure 2). While both pairs of genes had transcripts when the adult cuticle is being laid down, the two *CPLCG* genes have maximal transcript levels later than the *CPF* genes and their transcript levels were lower. The data had the same temporal pattern as our earlier studies, but there in young adults, *CPLCG4* was similar to *CPLCG3*[4] and *CPF4* was lower than *CPF3*[3]. Adult eclosion in *An. gambiae* is gated to occur after the dark period begins. Some pharate adults at P24 will be only a few hours away from eclosion, others will wait much longer. The animals we were comparing between our published work and this analysis were kept under different photoperiods and collected at different times of the day, so quantitative differences in relative transcript levels are not surprising, and indeed we observed this difference. (see Additional file 2).

### Transcript localization

**Figure 3:**
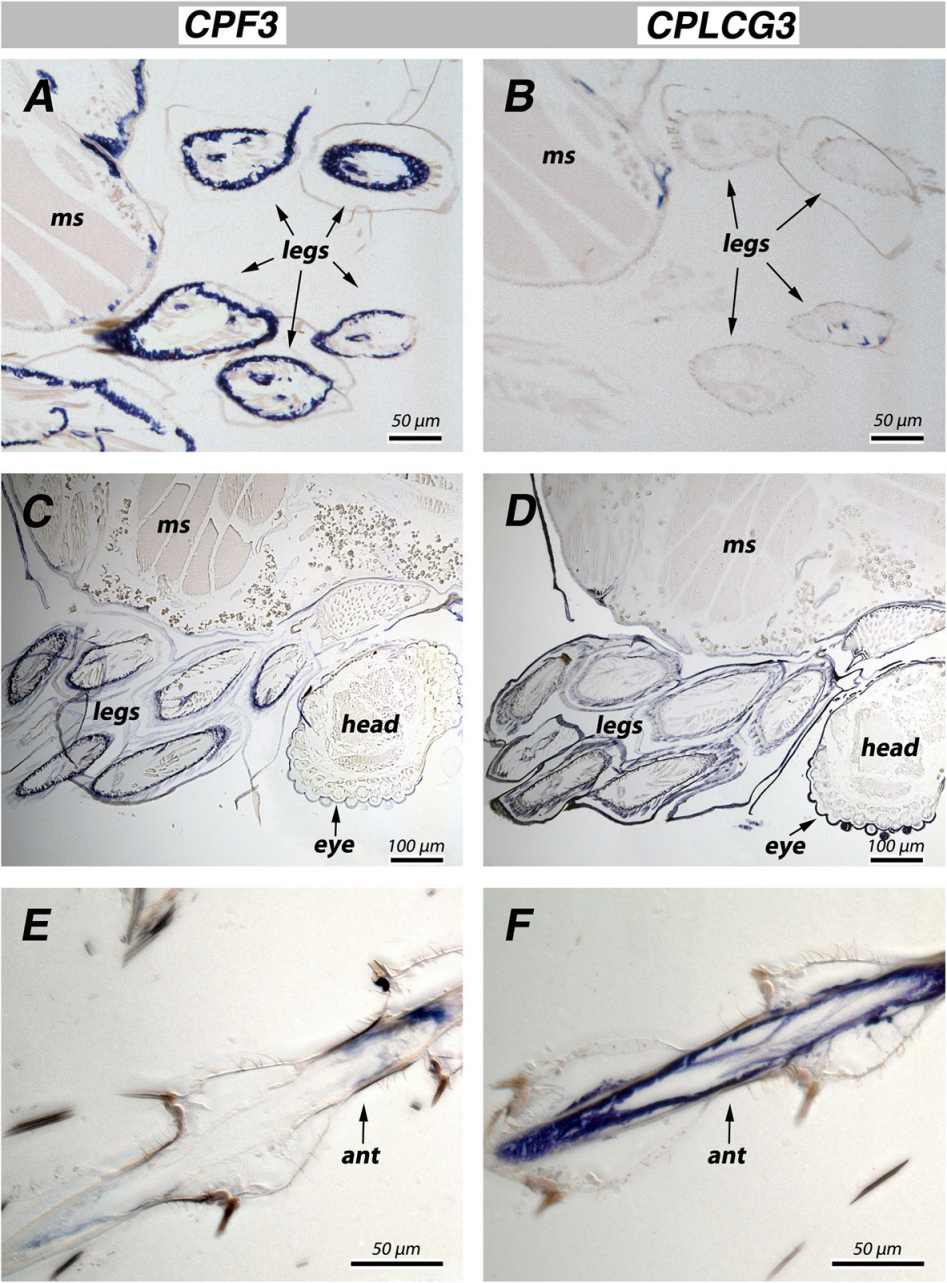
***In situ *hybridization to appendages of animals of different ages.** Most common is greater hybridization for *CPF3* than *CPLCG3* in P24 **(A, B)**. But occasionally there is similar hybridization of both at P24 **(C, D)**. After eclosion, *CPLCG3* is generally stronger in appendages than CPF3, here the antenna of a young adult male, 17–19 h post-eclosion **(E, F)**. ms, muscle; ant, antenna.

**Figure 4:**
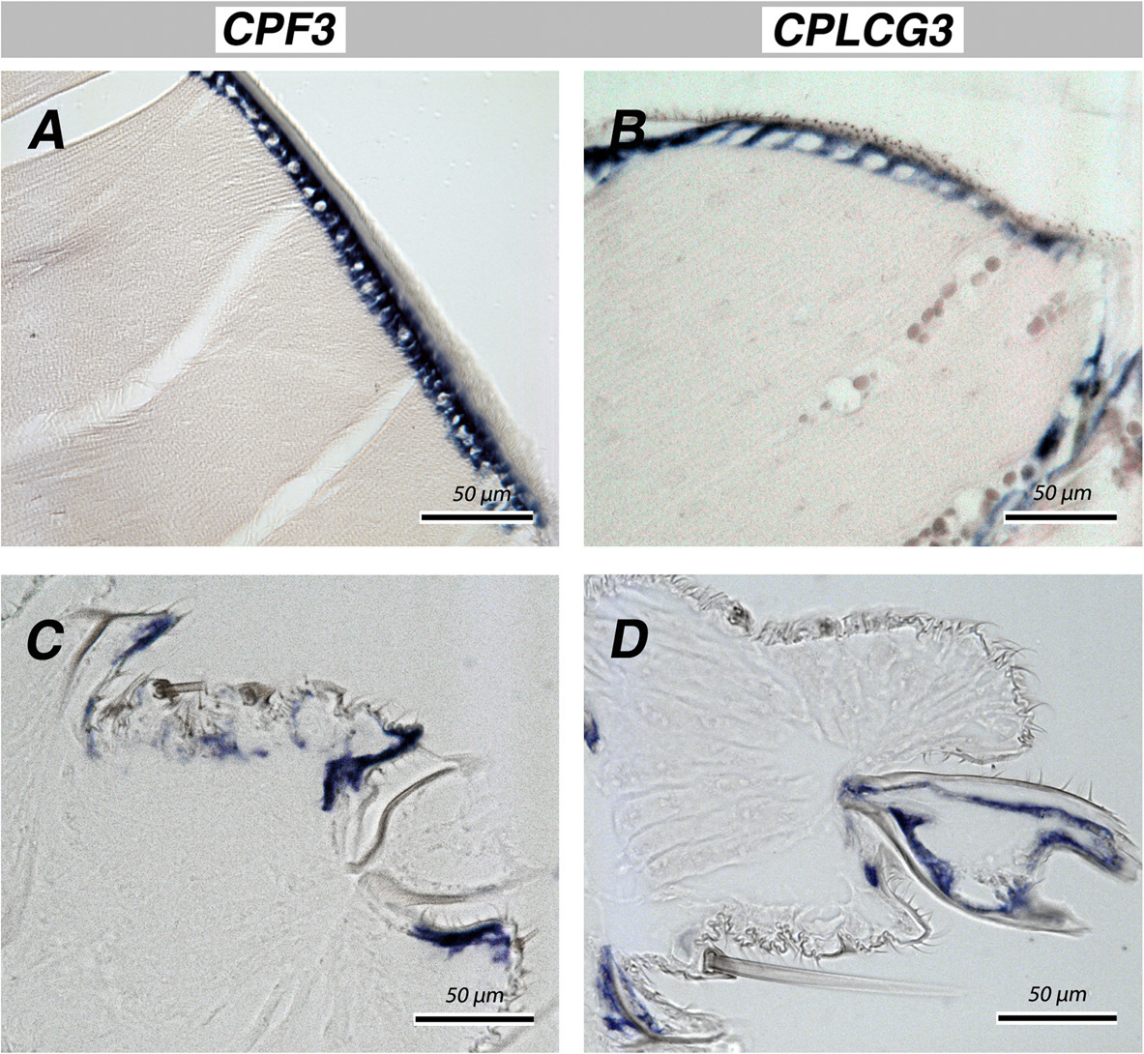
***In situ *hybridization to other regions.** Hybridization to muscle insertion zones for both *CPF3***(A)** and *CPLCG3***(B). (A)** maleP24, **(B)** female P24 **(C, D)** Hybridization to genitalia, **(C)** adult female 12 hr, **(D)** male P24.

Results from *in situ* hybridization are in accord with the mRNA temporal patterns, but reveal that the situation is more complex. The *CPF3* probe hybridized best in pharate adults (P24) (Figure 3A, C), while *CPLCG3* was most abundant in young adults (Figure 3F). Nonetheless, for some specimens, there was strong hybridization at the other stage (Figure 3D). Transcripts were also detected in other tissues. Transcripts from all four genes were present in the thorax where muscle and cuticle came in contact (muscle insertion zones) (examples in Figure 4A, B). In pharate adults of both sexes, all four genes had transcripts in the genitalia (examples in Figure 4C, D). None of the probes were detected in the eyes, with the exception of artifactual labeling of the acellular lens, a common problem with RNA probes which also frequently react with the old, acellular, pupal cuticle [11].

**Figure 5:**
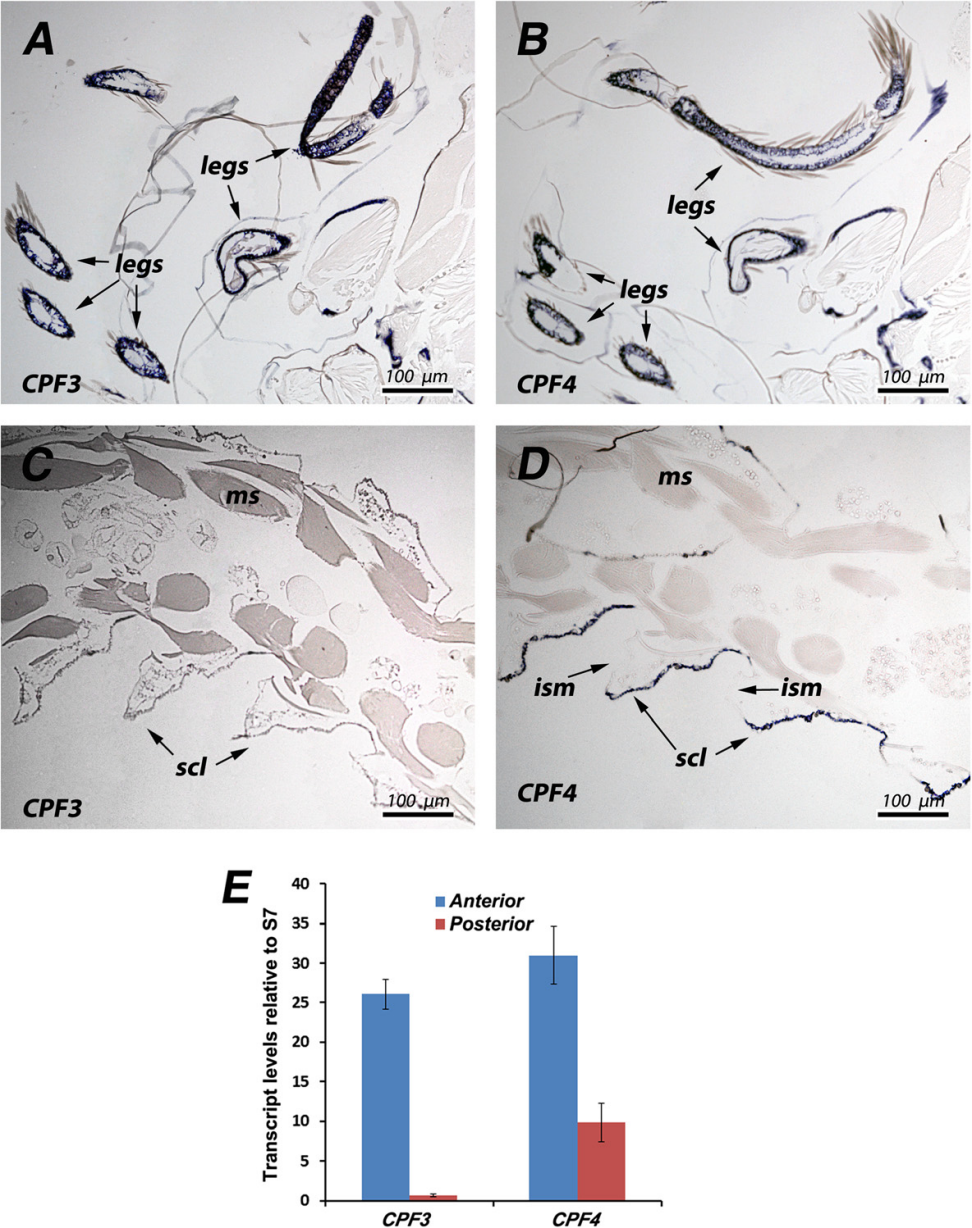
**Comparison of *CPF3 *and *CPF4 *expression in male pharate adults (P24).** Probes for both genes hybridize equally to the limbs **(A, B)**. However, only *CPF4* hybridizes to abdominal sclerites **(D)**, a location not seen with *CPF3***(C). (E)** RT-qPCR of *CPF3* and *CPF4* in pharate adult anterior (head and thorax) and posterior (abdomen) at P24. Standard errors are shown. ism, intersegmental membrane; scl, sclerites; ms, muscle.

While *CPF3* and *CPF4* had identical patterns of hybridization to appendages (Figure 5A, B), only the probe for *CPF4* reacted with the general epidermis of the pharate adult abdomen, and here, just the sclerites and not intersegmental membranes (Figure 5C, D). We carried out RT-qPCR for *CPF3* and *CPF4* on anterior (head and thorax) and posterior (abdomen) regions. Transcript levels were higher in the abdomen for *CPF4* than they were for *CPF3* (Figure 5E), a nice confirmation of the *in situ* results.

The presence of *CPLCG3* and *CPLCG4* in limbs is in accord with their role in insecticide resistance because those are the areas of the body that come in contact with insecticides that had been applied to surfaces. Likewise, the presence of *CPF3* in the genitalia might reflect a role in mating. But, the fact that the two *CPLCG*s were present in genitalia and both *CPF*s were present in limbs, complicates a simplistic story. Rather these results seem to suggest that CPF3/4 and CPLCG3/4 play complementary roles in formation of appendage cuticles.

### Western blot

**Figure 6:**
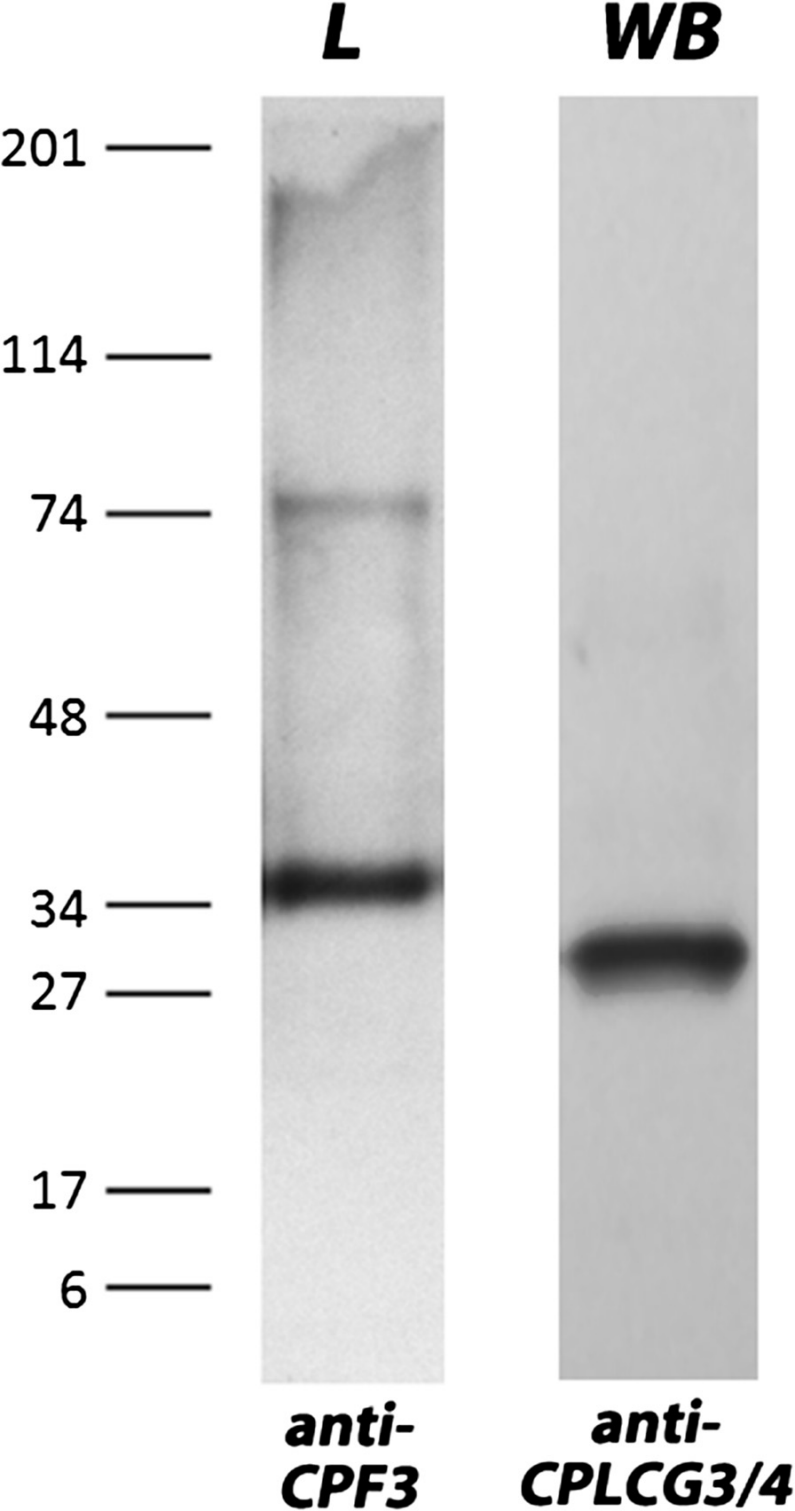
**Western blots of crude protein extracts.** Extracts from legs (L) and whole body (WB) treated with anti-CPF3 (diluted 1,000-fold) and anti-CPLCG3/4 (diluted 30,000-fold). Detection was with horseradish peroxidase-linked secondary antibodies, sheep anti-mouse (GE Healthcare, NA931) and goat anti-rabbit Sigma, A0545). Molecular weight marker is in kDa.

Western blot analysis of crude protein extracts from adult legs and bodies detected a strong band for CPF3 and CPLCG3/4 around 37 and 31kDa, respectively (Figure 6). A faint band around 74kDa was also detected for CPF3. The calculated molecular masses of the secreted proteins were: 12.49kDa for CPF3 and 10.75kDa for CPLCG3/4 (based on the average masses of the two proteins). Thus, it is possible that CPF3 forms trimers and a smaller amount of hexamers, because bands three- and six-times larger than the inferred molecular weight were detected. A trimer for CPLCG3, or CPLCG4 or a combination is also possible. Another contributing factor may be that the apparent different molecular masses reflect the previously described abnormal electrophoretic mobility of many cuticular proteins [13]. Unfortunately, the similar MWs of related CPLCG proteins (Additional file 4) means the single band found in the Western Blots does not guarantee that the antibody is solely recognizing CPLCG3/4.

### Immunocytochemistry

**Figure 7:**
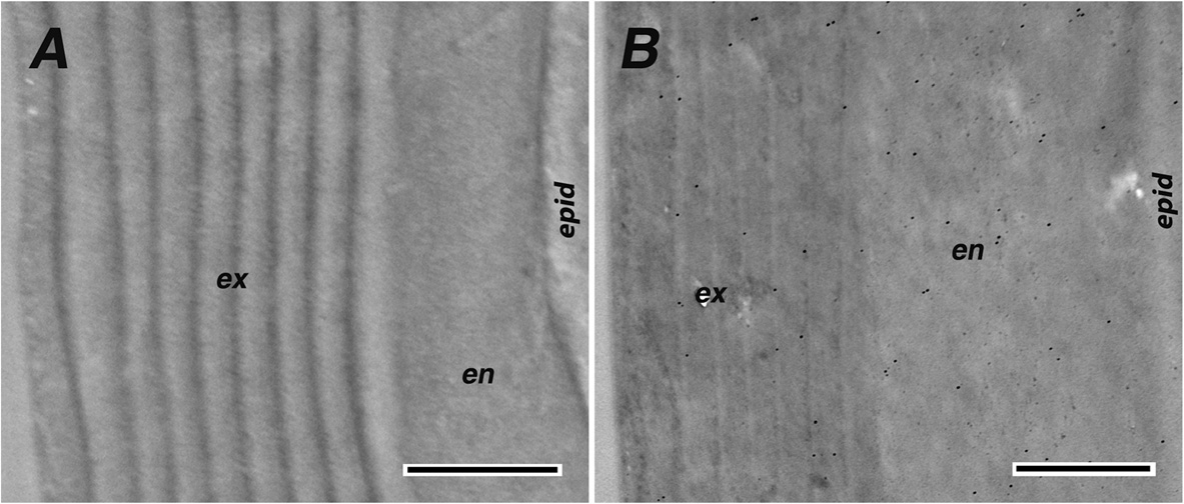
**Sections of 1-d-old mosquito legs treated with the pre-immune serum.** Preimmune serum was collected from the same animals used to raise the anti-CPF3 **(A)** and the anti-CPLCG3/4 **(B)**. Secondary antibodies were anti-mouse and anti-rabbit, respectively. ex, exocuticle; en, endocuticle; epid**,** epidermis. Scale bar=500 nm.

First we verified that the secondary antibodies that had been conjugated to colloidal gold did not, in themselves, react with components of the cuticle. We detected only an occasional dispersed gold particle when these secondary antibodies were tested on sections that had been incubated with the appropriate pre-immune serum (Figure 7).

**Figure 8:**
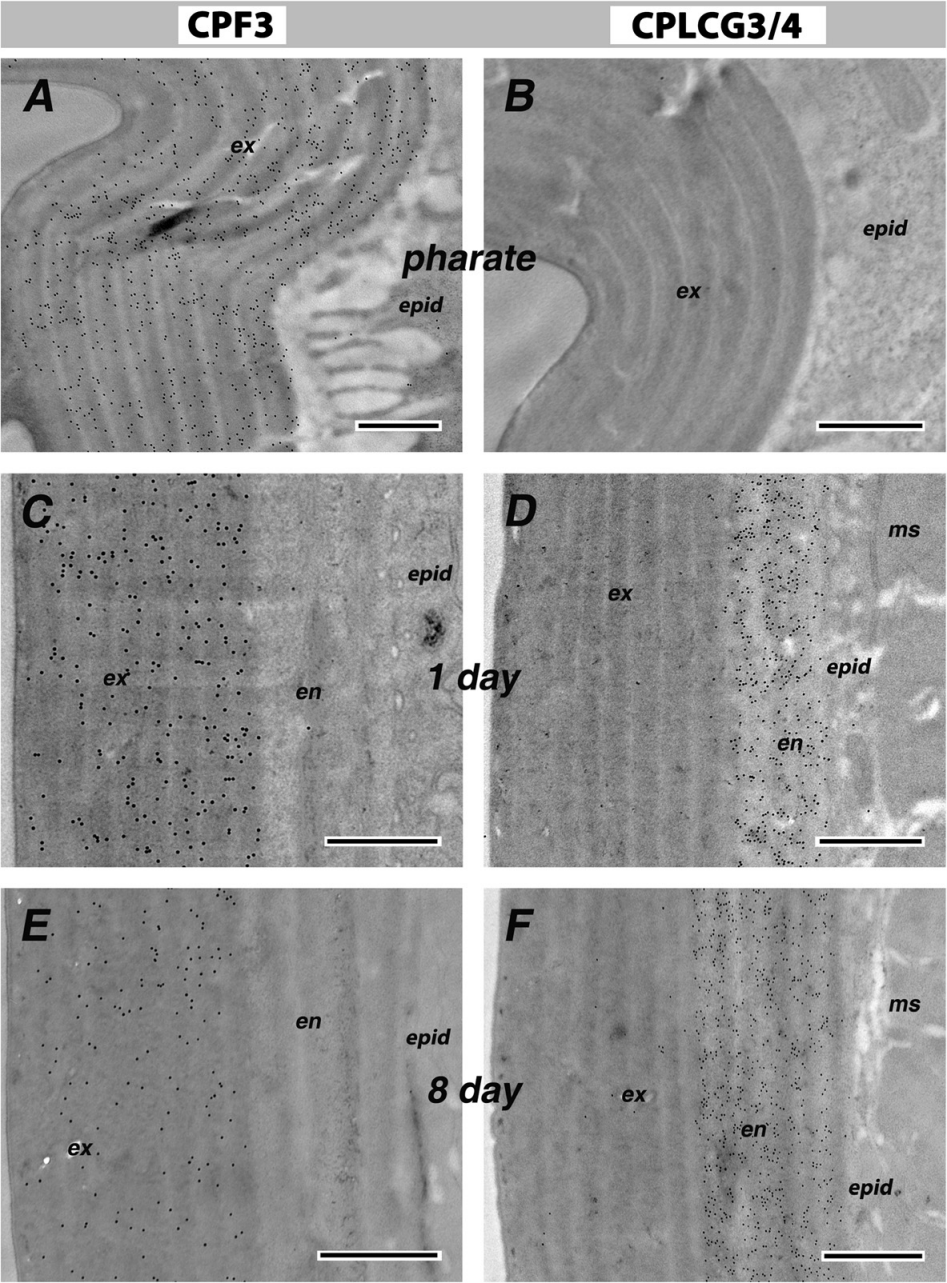
**Ultrastructural immunolocalization in leg cuticles.** Primary antibodies were raised against CPF3 (left panels) and CPLCG3/4 (right panels). **(A, B)** P24 pharate adults; **(C, D)** 1-d-old adults; **(E, F)** 8-d-old adults. Anti-CPF3 and Anti-CPLCG3/4 were detected by secondary antibodies, conjugated to 10 and 5 nm gold particles, respectively. ex, exocuticle; en, endocuticle; epid, epidermis; ms, muscle. Scale bar=500 nm. Apparent size of gold particles is dependent on focal plane.

CPF3 expression was detected throughout the cuticle at high levels in animals fixed at 24 h after pupation (pharate adults). At this stage, only the epicuticle and pre-ecdysial exocuticle are present (Figure 8A). After eclosion, four morphologically distinct cuticular layers can be identified (epicuticle, exo- and endo-cuticle and assembly zone). Here too, in 1-d-old adults, CPF3 was detected only in exocuticle (Figure 8C). Even in the oldest mosquitoes examined (8-d-old adults), CPF3 was restricted to exocuticle even though at this age, the endocuticle also appears lamellar (Figure 8E).

Togawa *et al*. [3] used the same assay that had been used to demonstrate chitin-binding by members of the CPR family [14,15] to learn if the CPF family had chitin binding properties. Neither recombinant CPF1 nor CPF3 bound chitin, although CPR21 tested at the same time did. Based on this result and the aggregation observed with the recombinant protein, they speculated that CPF3 might be located in the epicuticle, the layer of the insect cuticle that lacks in chitin [3]. A homology model of CPF3 indicated the presence of a pocket in a β-barrel structure [7]. Unlike a somewhat similar homology model for some CPR proteins [16], chitin could not be computationally docked in this pocket. Cassone *et al.*[5] had suggested that CPF3 might serve as a courtship modulator, thus explaining its different transcript levels in M and S incipient species. Papandreou *et al.*[7] thus computationally tested a *Drosophila* sex pheromone, 7(Z), 11(Z)-heptacosadiene and learned that it could be docked in the CPF3 pocket. Lacking any *Anopheles* pheromone to test, all this really revealed was that hydrocarbons could fit. Our data reveal that CPF3 is localized only in the exocuticle and thus is not well positioned to present a contact pheromone. So perhaps, CPF3 is just one of those cuticular proteins that fill spaces between the chitin binding proteins as suggested in a model of Andersen [17]. But an exciting possibility is that CPF3 holds hydrocarbons in the cuticle and its higher levels (if high transcript=high protein) in M than S, correlates provocatively with the greater desiccation resistance found in adults of the M form [18]. Indeed, the large differences in transcript levels between M and S fit better with a model where they are used for something less subtle than pheromone presentation, especially in a species where, to date, there is no evidence for a courtship pheromone.

**Figure 9:**
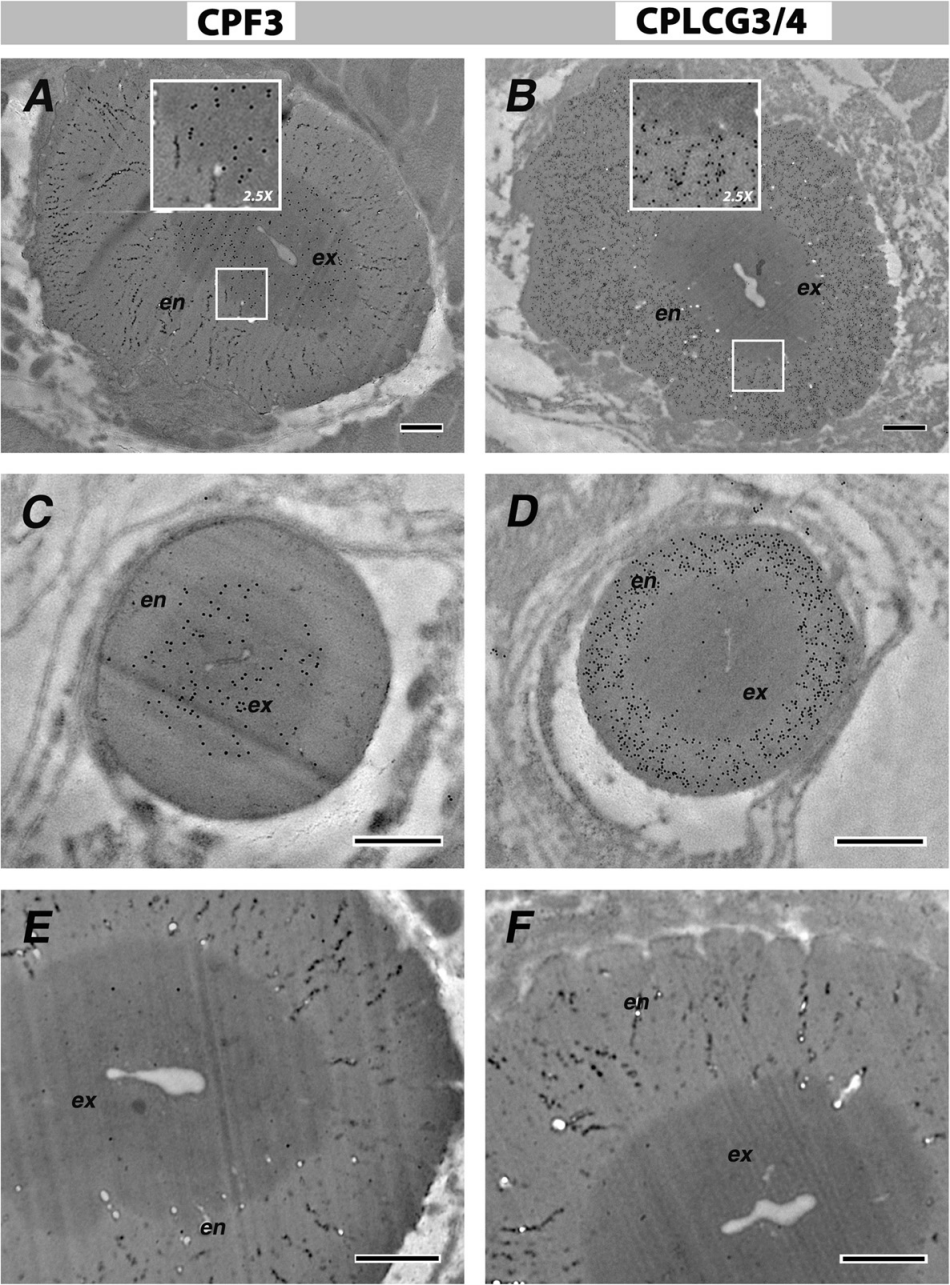
**Ultrastructural immunolocalization in leg apodemes of 1-d-old mosquitoes.** CPF3 (left panels) and CPLCG3/4 (right panels) are shown in the flexor tibiae apodeme cuticle **(A, B)**; and extensor tibiae apodeme **(C, D). (E, F)** Control sections treated with the appropriate pre-immune serum; note the granulated structures in the endocuticle. Sections were incubated with goat anti-mouse **(A, C, E)** or with goat anti-rabbit **(B, D, F)** secondary antibodies conjugated to 10- and 5-nm gold particles, respectively. ex, exocuticle; en, endocuticle Scale bar=500 nm.

CPLCG3/4 was not detected in the cuticle of pharate adults (Figure 8B). Rather, in contrast to the findings with CPF3, protein was found only in the endocuticle of both 1-d-old and 8-d-old adults (Figure 8D, F). CPF3 and CPLCG3/4 were also detected in the exocuticle and endocuticle, respectively, of *An. gambiae* flexor and extensor tibiae apodemes (Figure 9).

The predominant presence of CPLCG3 and CPLCG4 mRNAs in limbs and the abundance of the protein in limb cuticle correlates nicely with the >2-fold increased abundance of their transcript in pyrethroid resistance *An. gambiae*[9]. Furthermore, an earlier study found, with both microarray and RT-qPCR, that the CPLCG3 ortholog in *An. stephensi* was among the small number of transcripts that were more abundant in the insecticide-resistant form of that species [8]. Given that adult mosquitoes contact insecticides through their limbs, this would be a perfect site to have more abundant cuticular proteins underwriting a thicker cuticle. Wood *et al*. [19] have shown that pyrethroid resistant *An. funestus* do indeed have a thicker cuticle on their legs than sensitive forms and suggested that this might slow down penetration of the insecticide allowing more time for detoxification mechanisms to act. Higher levels of transcripts of CPs have been correlated with insecticide resistance in studies in other insects [20-22]. The older literature has examples of decreased penetration of labeled insecticides in resistant insects [23,24].

## Conclusions

These data provide additional information on why *An. gambiae* devotes almost 2% of its protein coding genes to structural cuticular proteins. Although CPF3/4 and CPLCG3/4 have overlapping periods of transcript expression and predominant transcript localization in the same tissues, appendages, their proteins are completely segregated in the cuticle. CPF3 is restricted to exocuticle and CPLCG3/4 is only found in the endocuticle. The presence of CPLCG3/4 in limbs correlates nicely with its role in insecticide resistance. The higher level of CPF3 transcripts in M than in S incipient species was once suggested to play a role in pheromone display. We now know that wing beat frequency is the major player in mate recognition [25], and while contact pheromones have not been ruled out, the localization of CPF3 in exo- and not epi-cuticle suggests that it is unlikely to be playing a role in mate recognition. But there remains a possibility that CPF3 contributes to the greater resistance to desiccation of the M form. The specific localization of CPs within the cuticle and the areas where CPF4 but not CPF3 probes hybridize in pharate adults are further indications that the multiplicity of CP genes must be, at least in part, because they are serving specific, but in some cases, overlapping functions.

## Competing interests

The authors declare that they have no competing interests.

## Authors’ contributions

LV and JHW conceived and designed the study, analyzed the data and drafted the MS. LV performed the immunological components. TWR carried out the RT-qPCR and helped with *in situ* hybridization. All authors read and approved the final version of the MS.

## Supplementary Material

Additional file 1 **DNA sequences and locations of primers and probes used for *in situ *** **hybridization.** (A) Nucleic acid sequences of *CPF3* and *CPF4.* (B) sequences of *CPLCG3* and *CPLCG4.* Shown are *in situ* primers (bold) and *in situ* probes (gray highlight), start (green) and stop (red) codons; CPLCG4 primers are in orange. Two different probes were used for *CPLCG3*, with primers indicated in black bold (for *CPLCG3*) and blue bold for *CPLCG3*-EA*.* Reverse primers are shown as the complement on the coding strand. Click here for file

Additional file 2 **Effect of photophase on transcript levels from P24 animals.** Female mosquitoes 24 h after pupation were harvested at different times relative to the start of the dark period. (AZT is Arbitrary Zeitgeber Time with time 0 the start of lights on.) For each CP transcript, means with different letters are statistically significant (p≤.05). (A) CPF3 and CPF4. (B) CPLCG3 and CPLCG4. (C) RpS7 threshold cycles for the data shown above. There are no significant differences between groups. RT-qPCR was performed with Bio-Rad’s CFX Connect Real Time System. We used three groups of three animals each for cDNA preparation for each condition. All values show mean±SEM. All reactions were carried out in triplicate (technical replicates) in a 15 μl reaction containing 3.75 μl of 1/100 diluted cDNAs (equivalent to starting with 5.6ng of total RNA), 250 nM of each primer, and 7.5 μl SsoAdvanced SYBR® Green Supermix (Bio-Rad). PCR conditions were 95°C for 2 min followed by 40 cycles of 95°C for 10s and 57°C for 30s. Click here for file

Additional file 3 Primers used for RT-qPCR. Click here for file

Additional file 4 **Potential off-target effects.** Each of the 14 aa peptides used to generate antibodies was submitted to BLAST (blastp) against the *Anopheles gambiae* proteome (PEST) and alignments produced are shown along with MWs and published data on transcript abundance obtained with RT-qPCR [3,4]. With the exception of CPLCG5, it is unlikely that other CPs would be recognized by the antibodies. CPLCG1 is expressed in many tissues including scales [11], so we know that it is not recognized by the antibody raised against CPLCG3/4. The RT-qPCR data come from measurements made with different conditions for animal rearing and transcript levels than those used for other data in this paper. Data for transcripts compared in the Table are based on the same conditions. Click here for file
