# Supplementary material for: Temporal and spatial expression of cuticular proteins of Anopheles gambiae implicated in insecticide resistance or differentiation of M/S incipient species
Source: Parasit Vectors. 2014 Jan 15;7:24. doi: 10.1186/1756-3305-7-24 (PMC3898775; doi:10.1186/1756-3305-7-24)
Supplement: Additional file 1 — DNA sequences and locations of primers and probes used for in situ hybridization. (A) Nucleic acid sequences of CPF3 and CPF4. (B) sequences of CPLCG3 and CPLCG4. Shown are in situ primers (bold) and in situ probes (gray highlight), start (green) and stop (red) codons; CPLCG4 primers are in orange. Two different probes were used for CPLCG3, with primers indicated in black bold (for CPLCG3) and blue bold for CPLCG3-EA. Reverse primers are shown as the complement on the coding strand. [file 1756-3305-7-24-S1.docx]

Additional File 1. DNA sequences and locations of primers and probes used for *in situ* hybridization

A.

CPF3 ----------------CTGAATTCAAAGCAGTCG----CATCGACGATTTCATTGACGTT

CPF4 **ATG**GCCTTCAAGTTTGTCATCCTTGCTGCACTCGTGGCCGCCGTCAGTGCCGGTGGACCG

* *** *** * ** * * * **

CPF3 ATCATTTATTCAGTTATTCTGTTCA----CATATTTCTCTGTTACA**ATG**TATCGTTTTG**T**

CPF4 GCCGCCTACTCGATTGCCGCGCCGAGCGCCGACTTCCACTCGGTCGGTGCGTCGCACGAG

* ** ** ** * * * ** * ** * ** ***

CPF3 **CGCTCTTTTT**---**GCTTTGGTTGCT**GTATCCCAAGCCGCCTACACTCTGAACCCAGCAGG

CPF4 CACACCGTCAAGGGCCTGTACGGCCAGAACGTGCTGTCCCAGTACTC-GAAGGCCGTCGA

* * * * ** * ** * * ** **** *** * * *

CPF3 CCCAACTTACGCA--GGTATTCACACTCCGGCCATTACCAGCCAGCAGTCTAATATCCTA

CPF4 CTCGGCGCACTCGTCGGTGCGTGTGCACAGCTCGCGCCTGAGCAACGACGGATACGCGTA

* * * ** * *** * * * * * ** * * * **

CPF3 CGCAGTTATGGAAACTTGGGACAG-ATCTCGACCTACTC-GAAGACCAT-CGATACTCCC

CPF4 CGCCGCCCCCGCCGTTAAGTACGCCGCCCCCGCCTACGCTGCCCATTATGCTGCCCCCGC

*** * * * * ** * * ***** * * * ** * * * *

CPF3 TACTCTTCAGTCAGCAA--ATCCGATGTGC-GAGTAAGCAATCCAGGACT-GGCGGTTGG

CPF4 CGTCCACTACCCGGCTGCCGCCCACTACGCTGCCCCCGCCGTCCACTACCCGGCTGCCGC

* * * ** ** * ** * ** **** ** *** * *

CPF3 CCATATCGCCGCTTC--TTATCCAC-ATCCAATCGCTGCTCCTGCTTATGGCCATGTCGG

CPF4 TCACTACGCCGCTCCGGCCGTCCACTACGCTGCCCACGCCCCGATCGTTAAGGCCGCCTA

** ******* * ***** * * * ** ** * * *

CPF3 TTATGCTGC-TACGGCACTT**AAGAATCCGGCACTACTGGGC**--**G**TTGCATATTCCGCTGC

CPF4 CCCCGCTGCCTACGCTGCCCCGCTGGCCTACAAGACTCCGCTGGCCGCTCCTGTAGCCGC

***** **** * ** ** *** ** * ** * ** **

CPF3 TCCAGCCGTTGCGCACATGACTTACAGCAATGGCCTCGGCATTAACTATGCCTGG**TAA**A-

CPF4 CGTGCACGGTGGATCCGTGGTCCAGTTCGCCGGT**CTCGGTGCCAGCTACGCCTG**G**TAA**GG

** ** * ** * * ** ***** * *** *********

CPF3 CATTGTTTTGGTCGAACTAAAACCATGTTTATCACCACCATCGATGCTAGTGAAAAACAA

CPF4 CCTCCCACTGACTGCATCGCACGCACTGATACCAACGATGATGATGATGATGTCGACGAC

* * ** * * * ** ** ** * **** * ** * *

CPF3 ACGTTCATCCTACAAATGCAAATGCAACACGGCCAATGAAACAAAAAGTCATA--GTCA-

CPF4 GACGTCGCACTAGCTCGGCAGTAGCAATAGCTTTACAGGAGAGTCAAGTCACACAGACAC

** *** *** **** * * * * ****** * * **

CPF3 --ACACAGTCAAACAATAAATAATAATTTATATTCGAATA-CAGTGTGTTGCTACG----

CPF4 ACACACAGACAGATAGCAAAAAACAGTTAGCAGCTTAGTAACTAAGTTCTTCTTAGCAAC

****** ** * * *** ** * ** * * ** * ** * ** *

CPF3 ---TGTGTACTCGGGGGCGAGCACTACGCCAACTAAGCGGCCCC--------------

CPF4 CACTGTGCATTTCGGGTGAA**CAGCCCCCTCGAGCAAGGA**TGCGAATAAATGTGAGACA

**** * * *** * * * * * *** *

B.

CPLCG3 **ATG**AAGTG**CATGGTAGCTGCAGTCATC**TTGGCCCTGGCCGTCGTTTCGGAAGCCGGACTC

CPLCG4 **ATG**AAGGTCGCCGTTGTTGCCGTTGTTCTCGCCCTTGCCGTCGTCTCGGAGGCTGGAGTG

****** * ** * *** ** * * ***** ******** ***** ** *** *

CPLCG3 CTGCCGTACGGTGGCTGGCCTTATGGTCATCTGGCTGCCCCGACCGTCATCCAGTCGAAT

CPLCG4 CTTCCGT---GGGGCTGGCCATACG-----CTGGACTTCCGGCCGCTTACCCAGTTGC--

** **** * ******** ** * **** ** * * * * ***** *

CPLCG3 GTGCTGGCCCACCCGTACGGTGCGATCTCGCACGCCGCCATCCACGCTGCTTACGCTCCG

CPLCG4 -TGCGTGGCCACCGGCA-------------------GCCATCCATGCCGCCTACCCAGCG

*** * ***** * * ******** ** ** *** * **

CPLCG3 CATGCTGCCATCCTGGCCGCGCCGCACGCTGCCATTCTGGCCGCTCCACATGCTGCCATC

CPLCG4 TACGCT--CACCATGGC-------------GCTTATCTGGCCGCCCCGCACGCTGCCATC

* *** ** * **** ** ********* ** ** *********

CPLCG3 TTGGCCGCTCCGCATGCCCCAGCTGCGTCGGTCGCTCACC**ATGCTGGAGTCGTCCCT**GGA

CPLCG4 CTGGCCGCTCCTCATGCCCCAGCTGCCTCGGTCGCTCACCATGCCGGTGTTGTCCCTGGA

********** ************** ***************** ** ** *********

CPLCG3 GCCACCTCCGTCACTGCTACCCGAGGTGCTGTGCATGTCGCGCCCCTGCCTGGACATGCC

CPLCG4 GCCACCTCGGTCACTGCCACCCGTGGTGCTGTGCATGTCGCGCCCCTGCCTGGACATGCC

******** ******** ***** ************************************

CPLCG3 GTCTCCCAGCAGCAGCTGAACCTGGCCCCGGCCCCTGGCACGCTC**TAA**GCCAT**CCCATCT**

CPLCG4 GTCTCCCAGCAGCAGCTGAACCTGGCCCCGGCCCCGGGAACCATC**TAA**G---TCCGAGTA

*********************************** ** ** ****** *** *

CPLCG3 **CCTAGTCAGTGTG**CCCCCGGTTCGGGTGCTCTGGAACATTCTATCTCTTACCATCTGTCG

CPLCG4 C--ACCCAGACCG**CACCCAAAC**--**ATTCAACTGGA**ACTCATCATCCCACACCGTGTGCAA

* * *** ** *** * ******* *** * *** * **

CPLCG3 TCTATCGCTATATTGTTCAAAAGTTACGCTATCCCGATTTGAATGGTAGAGTTACATCGT

CPLCG4 AT--CCGCTGAAC-------AAGTGATAACATACTCATT--AGCTGTTAAGT-AGTTCGT

**** * **** * ** * *** * ** *** * ****

CPLCG3 TCGACCCCC**ACTT**-**CATTCGGACCTGGAAC**GCGCGTGTGT-GTGTTTGAATAAAACGAAC

CPLCG4 TCGTTTGCGAT**CTGTTTTCGTTTCCACTGCA**TTCACGCAAAGTGCTTGAATAAAGGCAAT

*** * * * **** * * * * *** ********* **

CPLCG3 AAACCAAATTTGGTTTCATTTTTCTCATT

CPLCG4 AATGCAAAGC-------------------

** ****
