# Supplementary material for: Temporal and spatial expression of cuticular proteins of Anopheles gambiae implicated in insecticide resistance or differentiation of M/S incipient species
Source: Parasit Vectors. 2014 Jan 15;7:24. doi: 10.1186/1756-3305-7-24 (PMC3898775; doi:10.1186/1756-3305-7-24)
Supplement: Additional file 2 — Effect of photophase on transcript levels from P24 animals. Female mosquitoes 24 h after pupation were harvested at different times relative to the start of the dark period. (AZT is Arbitrary Zeitgeber Time with time 0 the start of lights on.) For each CP transcript, means with different letters are statistically significant (p≤.05). (A) CPF3 and CPF4. (B) CPLCG3 and CPLCG4. (C) RpS7 threshold cycles for the data shown above. There are no significant differences between groups. RT-qPCR was performed with Bio-Rad’s CFX Connect Real Time System. We used three groups of three animals each for cDNA preparation for each condition. All values show mean±SEM. All reactions were carried out in triplicate (technical replicates) in a 15 μl reaction containing 3.75 μl of 1/100 diluted cDNAs (equivalent to starting with 5.6ng of total RNA), 250 nM of each primer, and 7.5 μl SsoAdvanced SYBR® Green Supermix (Bio-Rad). PCR conditions were 95°C for 2 min followed by 40 cycles of 95°C for 10s and 57°C for 30s. [file 1756-3305-7-24-S2.docx]

Additional File 2. Effect of Photophase on transcript levels from P24 animals.

**C.**

| Sample Description | RpS7 Threshold Cycle |
| --- | --- |
| 12:12, AZT=8.5 | 22.46 ± 0.15 |
| 12:12, AZT=11.7 | 22.63 ± 0.14 |
| 16:8, AZT=10.5 | 22.86 ± 0.12 |
| 16:8, AZT=11.8 | 22.81 ± 0.21 |
