# Supplementary material for: Temporal and spatial expression of cuticular proteins of Anopheles gambiae implicated in insecticide resistance or differentiation of M/S incipient species
Source: Parasit Vectors. 2014 Jan 15;7:24. doi: 10.1186/1756-3305-7-24 (PMC3898775; doi:10.1186/1756-3305-7-24)
Supplement: Additional file 3 — Primers used for RT-qPCR. [file 1756-3305-7-24-S3.docx]

| Gene |  | RT-qPCR Primer Sequence |
| --- | --- | --- |
| RpS7 | F | GTGAGGTCGAGTTCAACAACAAGAA |
|  | R | GGCACCGGCACGTAGATGA |
| CPF3 | F | CCACCATCGATGCTAGTGAAAAACAAA |
|  | R | TTTCATTGGCCGTGTTGCATT |
| CPF4 | F | TCGGCAGTAGCAATAGCTTTACAGGA |
|  | R | GGGGCTGTTCACCCGAAATG |
| CPLCG3 | F | TCCCATCTCCTAGTCAGTGTGC |
|  | R | TGAAGTGGGGGTCGAACGATG |
| CPLCG4 | F | CCCAGACCGCACCCAAACAT |
|  | R | TGTTCAGCGGATTTGCACACG |
