# Supplementary material for: Temporal and spatial expression of cuticular proteins of Anopheles gambiae implicated in insecticide resistance or differentiation of M/S incipient species
Source: Parasit Vectors. 2014 Jan 15;7:24. doi: 10.1186/1756-3305-7-24 (PMC3898775; doi:10.1186/1756-3305-7-24)
Supplement: Additional file 4 — Potential off-target effects. Each of the 14 aa peptides used to generate antibodies was submitted to BLAST (blastp) against the Anopheles gambiae proteome (PEST) and alignments produced are shown along with MWs and published data on transcript abundance obtained with RT-qPCR [3,4]. With the exception of CPLCG5, it is unlikely that other CPs would be recognized by the antibodies. CPLCG1 is expressed in many tissues including scales [11], so we know that it is not recognized by the antibody raised against CPLCG3/4. The RT-qPCR data come from measurements made with different conditions for animal rearing and transcript levels than those used for other data in this paper. Data for transcripts compared in the Table are based on the same conditions. [file 1756-3305-7-24-S4.docx]

Additional File 4. Potential off target reactions.

| Top Blast hits  (MW) | E-value | Match | % identity in matched region | Transcript levels  (normalized to S7) from [3,4] | |
| --- | --- | --- | --- | --- | --- |
|  |  |  |  | P24 | A 0-12 |
| CPLCG3 (11,432) | 7.00E-08 | 14/14 | 100% | 0.78 | 1.81 |
| CPLCG4 (10,073) | 6.00E-07 | 13/13 | 100% | 2.61 | 7.35 |
| CPLCG5 (11,941) | 3.00E-06 | 13/14 | 93% | 3.66 | 6.34 |
| CPLCG14 (12,234) | 3.00E-05 | 12/13 | 92% | 0.05 | 0.09 |
| CPLCG1 (11,585) | 4.00E-04 | 11/12 | 92% | 0.02 | 3.60 |
| CPLCG15 (11,173) | 4.00E-04 | 11/13 | 85% | 0.00 | 0.00 |
| CPLCG23 (10,509) | 0.022 | 10/12 | 83% | 0.00 | 0.00 |
|  | | | | | |
| CPF3 (12,493) | 5.00E-08 | 14/14 | 100% | 239 | 3.4 |
| CPF2 (28,831) | 1.00E-04 | 11/14 | 79% | 0.45 | 0.006 |
| CPF1 (22,845) | 0.028 | 9/14 | 64% | 0.03 | 0.002 |
| CPR132 (41,960) | 1.8 | 10/17 | 59% | Exclusively in eye | |

| Alignments of sequences with similar peptides to those used for EM immunolocalization | |
| --- | --- |
| G3 PEPTIDE SQQQLNLAPAPGTL  SQQQLNLAPAPGTL  CPLCG3 SQQQLNLAPAPGTL | G3 PEPTIDE SQQQLNLAPAPGTL  SQQQLNLAPAPGT  CPLCG4 SQQQLNLAPAPGTI |
| G3 PEPTIDE SQQQLNLAPAPGTL  SQ QLNLAPAPGTL  CPLCG5 SQKQLNLAPAPGTL | G3 PEPTIDE SQQQLNLAPAPGTL  QQ LNL PAPGTL  CPLCG14 NQQSLNLEPAPGTL |
| G3 PEPTIDE SQQQLNLAPAPGTL  SQ QLNLAPAPG  CPLCG1 SQTQLNLAPAPGSE | G3 PEPTIDE SQQQLNLAPAPGTL  QQ LNL PAPGTL  CPLCG15 NQQSLNLEPAPGTL |
| G3 PEPTIDE SQQQLNLAPAPGTL  SQQQLNL APG  CPLCG23 SQQQLNLEAAPGSD |  |
|  | |
| CPF3 PEPTIDE KTIDTPYSSVSKSD  KTIDTPYSSVSKSD  CPF3 KTIDTPYSSVSKSD | CPF3 PEPTIDE KTIDTPYSSVSKSD  K +DTPYSSV KSD  CPF2 KAVDTPYSSVRKSD |
| CPF3 PEPTIDE KTIDTPYSSVSKSD  K +DT +SSV KSD  CPF1 113 KAVDTAFSSVRKSD | CPF3 PEPTIDE KTIDT---P-YSSVSKSD  TIDT P Y SVS D  CPR132 DTIDTIIAPAYTSVSRVD |
